# Inhibition of gastric acid secretion with omeprazole affects fish specific dynamic action and growth rate: Implications for the development of phenotypic stomach loss

**DOI:** 10.3389/fphys.2022.966447

**Published:** 2022-09-27

**Authors:** Kelsy Moffatt, Mark Rossi, Edward Park, Jon Christian Svendsen, Jonathan M. Wilson

**Affiliations:** ^1^ Wilfrid Laurier University, Waterloo, Canada; ^2^ Technical University of Denmark, National Institute of Aquatic Resources, Lyngby, Denmark; ^3^ CIIMAR University of Porto, Matosinhos, Portugal

**Keywords:** *Oreochromis niloticus*, specific growth rate, metabolic rate, H^+^/K^+^-ATPase, stomach

## Abstract

An acid-secreting stomach provides many selective advantages to fish and other vertebrates; however, phenotypic stomach loss has occurred independently multiple times and is linked to loss of expression of both the gastric proton pump and the protease pepsin. Reasons underpinning stomach loss remain uncertain. Understanding the importance of gastric acid-secretion to the metabolic costs of digestion and growth will provide information about the metabolic expense of acid-production and performance. In this study, omeprazole, a well characterized gastric proton pump inhibitor, was used to simulate the agastric phenotype by significantly inhibiting gastric acidification in Nile tilapia. The effects on post-prandial metabolic rate and growth were assessed using intermittent flow respirometry and growth trials, respectively. Omeprazole reduced the duration (34.4%) and magnitude (34.5%) of the specific dynamic action and specific growth rate (21.3%) suggesting a decrease in digestion and assimilation of the meal. Gastric pH was measured in control and omeprazole treated fish to confirm that gastric acid secretion was inhibited for up to 12 h post-treatment (*p* < 0.05). Gastric evacuation measurements confirm a more rapid emptying of the stomach in omeprazole treated fish. These findings reinforce the importance of stomach acidification in digestion and growth and present a novel way of determining costs of gastric digestion.

## Introduction

The stomach is a highly conserved vertebrate organ that provides a number of key advantages in the digestion of nutrients (Wilson and Castro 2010). Emerging in an elasmobranch ancestor approximately 350 million years ago (Koelz 1992), the stomach is defined by the production of hydrochloric acid (HCl) and pepsinogen (Smit 1968) by specialized oxynticopeptic cells. Pepsinogen, the inactive zymogen, is cleaved by HCl to produce activated pepsin, a proteolytic digestive enzyme (Sanny et al., 1975; Kageyama 2002). The stomach plays a key role in the breakdown and digestion of many food components including protein (Barrington 1942; Bakke et al., 2010), animal exoskeletons, plant cell walls (Lobel 1981), and solubilization of elements including phosphorus (Sugiura 2006) and calcium (Koelz 1992). Protein denaturation relies heavily on low pH in the stomach and phosphorus solubilization at low pH makes different supplemental forms more bio-available (Cho and Bureau 2001).

Despite the clear advantages of acid-peptic digestion, paradoxically many teleost species are agastric due to secondary loss of the acid-peptic digestive phenotype (Wilson and Castro 2010). Notably, these loss events result in not only phenotypic loss of the stomach, but also the loss of key stomach genes from the genome indicating a permanence of this loss (Castro et al., 2014). The key genes in this loss are the *atp4a* and *atp4b* that encode the respective α and β subunits of the gastric proton pump (H^+^/K^+^-ATPase), as well as various pepsin encoding genes (Castro et al., 2012, 2014). The gastric proton pump that produces the characteristic HCl in the stomach lumen is energetically expensive, as it relies on ATP-hydrolysis for ion movement, and there are costs associated with the maintenance of protective mucus and the neutralizing of the acid in the small intestine by bicarbonate. In vertebrate species that maintain gastric acid production, the energetic costs of gastric acid secretion are outweighed by the increased efficiency of nutrient utilization, particularly protein via pepsin digestion, and thus enhanced growth rates. It is hypothesized that the costs of gastric acidification might outweigh the benefits under some circumstances, ultimately resulting in the loss of these genes over time.

To better understand the role of the stomach in digestion, metabolic costs of acid-peptic digestion need to be determined. The increase in metabolic rate observed after the ingestion of a meal has been documented in many animals (Secor 2009). Termed the specific dynamic action (SDA) or the thermic effect of feeding, this increase in energy expenditure is thought to be the “cost of digestion” from mastication and secretion to digestion and assimilation (Secor 2009; Chabot et al., 2016). SDA represents the metabolic rate associated with feeding, which exceeds the standard metabolic rate (SMR), a basic maintenance requirement measured as the minimum rate of oxygen consumption of non-feeding, unstressed animals at rest, below which physiological function is impaired (Priede 1985). SDA has been previously used in fin fish aquaculture to identify optimal meal sizes, meal composition, ideal water temperature, and water pH that minimizes the SDA response, allowing for increased energy to be allocated to growth (e.g., Jobling 1981; Chakraborty et al., 1995; Tirsgaard et al., 2015). Despite these studies focussing on optimizing feeding responses, there have been limited efforts to determine the metabolic cost of gastric digestion, with one earlier study in Burmese python (Secor 2003), which estimated the stomach contributed 55% of SDA for a meal equivalent to 25% of body mass (BM) although this high value is disputed (see Wang and Rindom 2021). However, in order to isolate the direct metabolic costs of stomach acidification, the proton pump inhibitor omeprazole, can be used to inhibit this acid production (Andrade et al., 2004; Wood et al., 2009; Schubert 2017). Omeprazole provides a tool for unique comparisons of growth rates and SDA patterns in animals by inhibiting stomach acidification to create a pharmacological knock-down of gastric-acid secretion thereby mimicking the agastric condition.

Omeprazole, a benzimidazole derivative, acts to prevent gastric acid secretion by binding cysteine residues on the luminal side of the gastric proton pump H^+^/K^+^-ATPase, irreversibly blocking pump function (Lindberg et al., 1987). Omeprazole is a highly specific drug that works only in the acidic environment of the canaliculus of the acid-secreting oxynticopeptic or parietal cells making it an excellent candidate for analyzing energy usage during acid-digestion (Lindberg et al., 1986; Morii et al., 1989). Omeprazole is widely used in human medicine to reduce stomach acid production (McTavish et al., 1991) and has been used in spiny dogfish (25 mg kg^−1^ BM; *Squalus acanthias,* Wood et al., 2009) and common boa (22 mg kg^−1^ BM; *Boa constrictor,* Andrade et al., 2004) to study the post-feeding alkaline tide. The former study confirmed the acid-reducing effect of omeprazole in fish and highlighted future applicability of the drug. Omeprazole has also been used to study extragastric (gill and kidney) H^+^/K^+^-ATPase in Nile tilapia (*Oreochromis niloticus*) (Barnawi et al., 2020). In humans, omeprazole has been shown to delay overall assimilation of protein and cause potential protein malabsorption (Evenepoel et al., 1998). However, from a nutritional point of view in humans its impacts are likely negligible (Evenepoel et al., 1998) although not without risks in vulnerable groups (Heidelbaugh 2013).

In the present study, omeprazole was used to reveal the contribution of gastric acidification to patterns of the SDA and growth in the Nile tilapia (*O. niloticus*), a fish with a highly acidic stomach (pH 2.0; Moriarty 1973) and rapid growth rate with significant commercial importance in aquaculture (Maclean et al., 2002). A multi-experimental approach was used to confirm inhibition of gastric acid secretion and examine the effects of this treatment on growth and digestion. Four experiments are outlined: 1) The effects of omeprazole on gastric pH were measured to confirm inhibition of gastric acid secretion. 2) Next, the SDA was measured to examine the overall metabolic costs of digestion with inhibited gastric acid secretion. To better understand the effects of omeprazole on SDA and long-term effects of the treatment 3) growth rate was assessed over an eight-week feeding trial and 4) gastric evacuation rate determined. With respect to SDA, we predicted a decreased contribution of the H^+^/K^+^-ATPase to SDA peak ṀO_2_ and increase in time to peak of the post-prandial metabolic response because acidification occurs earlier in the digestive process. As inhibition of gastric acidification by omeprazole should reduce activation of pepsin and thus protein digestion, we also predicted reduced protein breakdown which should translate into a prolonged SDA duration and a decrease in growth rate. With the delay in acidification, we predicted a decrease in gastric evacuation rates with omeprazole treatment since acidification would take longer.

## Materials and methods

### Animals

Juvenile male Nile tilapia (*O. niloticus*) were obtained from Sandplains Aquaculture (Mossley, ON) and acclimated in 500 L recirculation tanks with mechanical and biological filtration, aeration, and 20% daily water changes in the Wilfrid Laurier University animal care facility. Water was prepared by mixing two parts reverse osmosis (RO) water with one-part city of Waterloo dechlorinated tap water to reduce water conductivity and hardness. The tilapia water had a final conductivity of approximately 300 μS cm^−1^, pH 7.7, Na^+^ 2.86 mM Cl^−^ 0.85 mM, Ca^2+^ hardness 300 mg L^−1^ CaCO_3_ and alkalinity of 80 mg L^−1^ CaCO_3_. Temperature was maintained at 26°C using a thermostat (InkBird Tech, Shenzhen, PRC) and immersion heater (300W Eheim Gmbh, Deizisau, Germany) in each tank. The light regime was 12 h light: 12 h dark. All experiments were conducted under the Canadian Council for Animal Care guidelines using protocols approved by the Laurier Animal Care Committee (AUP R14002 and R18003).

### Preparation of control and omeprazole dosed diets

In all trials, the control and omeprazole treated diets were prepared using the same procedure. Omeprazole and control diets were prepared using a commercial balanced diet (Bluewater Feed Company, Desboro, ON, Canada) formulated with the guaranteed analysis presented in Table 1. Omeprazole was added to the treated diet at a dose of 25 mg kg^−1^ BM day^−1^ (Wood et al., 2009) based on either a 1% or 2% BM daily ration by initially dissolving it in 95% ethyl alcohol (EtOH) and spraying it onto the feed. The feed was then air dried to allow for EtOH evaporation and was stored at 4°C in sufficient amounts to feed fish for 1 week. EtOH was added to the control diet and was air-dried to control for any effects of residual EtOH on gastric acidification, energy expenditure and/or growth.

**TABLE 1:** Guaranteed analysis of the commercial balanced diet used. Bluewater Feed Company Trout 2M 50-19.

Crude protein	Min	50.0%
Crude Fat	Min	19.0%
Crude Fiber	Max	2.3%
Sodium	Actual	0.4%
Calcium	Actual	1.5%
Phosphorus	Actual	1.2%
Vitamin A	Min	4,000 IU/kg
Vitamin D	Min	2,500 IU/kg
Vitamin E	Min	131 IU/kg

### Experimental procedures to measure gut pH

Understanding the extent to which omeprazole affects gastric pH was critical for this study. The pH trial was conducted using two groups of 30 Nile tilapia (30.4 ± 5.9 g) randomly assigned to one of the treatment groups. Feed (sham control and omeprazole) was given at a set time each day and daily ration was recorded. After 2 weeks on the treatment rations, fish were removed from the tanks at 3, 6-, 12-, 24- and 48-h post-feeding for determination of gastric and anterior intestinal pH (n = 6). Serial dissection was chosen for this experiment as the ‘T’ shape and relatively small size of the stomach (Morrison and Wright 1999; 1.4% (v:w) BM for a mean fish mass of 143 g, n = 34, unpublished data) makes *in vivo* pH measurement potentially harmful and stressful to the fish.

At each time point, the tilapia were euthanized with an overdose of MS222 (1:5000 w:v; Syndel, Nanaimo BC Canada) buffered with NaHCO_3_ followed by spinal transection, and fish mass and standard length were recorded. Glass combination pH electrodes (Biotrode, Hamilton) and Radiometer PHM85 pH meters were used to measure pH. The pH was measured first in the full excised stomach including chyme, the emptied stomach, and finally in the separated stomach contents. The anterior intestine was also removed and ligated 3 cm from the pyloric sphincter to form a gut sac. The pH of the chyme in this sac was recorded. The pH electrodes were rinsed with deionized water after each measurement and calibration was checked twice daily using precision standards (Hanna Instruments, Woonsocket RI). Negligible electrode drift was observed. The pH of the feed was determined in a slurry created by mixing 1 g of feed pulverized with a mortar and pestle in 10 ml of Milli Q water at ambient temperature (24°C).

The buffer capacity of the feed was also determined by manually titrating this 1 g pellet mixture using 1 N HCl and the same pH system described above with a Radiometer TTA80 titration assembly. The resulting titration curve (Supplementary Figure S1) was used to calculate the amount of acid required to acidify 1 g of pellets from its original pH based on the full stomach pH measurement over the 0–3 h and 0–6 h time intervals. Two linear regressions were fitted to the data covering the pH ranges of 6.2–5.0 [y = −0.0120 x + 6.238. *r* ^2^ 0.97] and pH 5.0–2.0 [y = −0.00251x+5.346 *r* ^2^ 0.99]. Gastric acid secretion is expressed as µEq H^+^ g^−1^ feed h^−1^.

### Respirometry

Oxygen consumption rates (Ṁ_O2_; µmol O_2_ min^−1^ kg^−1^) in Nile tilapia were determined using automated intermittent flow respirometry (Steffensen 1989). Respirometry was performed using a set of four 1-L respirometry chambers, each with a galvanic oxygen electrode connected to a 4-channel oxygen analyzer (AMP-DAQ4) and flush and recirculation pumps controlled through a DAQ-M unit using AutoResp™ software (version 2.2.0; Loligo^®^ Systems Viborg Denmark). The setup corresponded to previous studies measuring Ṁ_O2_ in fish (Tirsgaard et al., 2015; Rosewarne et al., 2016). The galvanic oxygen electrodes were calibrated using a 1g L^−1^ solution of sodium sulfite in water (0%) and in air (100% air saturation). ṀO_2_ was measured every 10.5 min cycling between 4 min flush, 0.5 min wait, and 6 min measurement periods. Specifically, Ṁ_O2_ was calculated using the rate of the declining oxygen content during the measurement period (Svendsen et al., 2015). The associated *R* ^2^ values above 0.95 were used for Ṁ_O2_ measurements. To avoid physiological effects of hypoxia, water oxygen levels were maintained above 80% air saturation (O_2sat_) during the measurement period, similar to previous respirometry studies (Tirsgaard et al., 2015; Baktoft et al., 2016). The oxygen consumption rates were used as a proxy for aerobic metabolic rate and thus energy use (Nelson 2016; Schwartzbach et al., 2020). The respirometry systems were submerged in a 450 L trough tank of circulating, filtered and aerated water kept at a consistent 26°C using a temperature regulator (TMP-REG, Loligo^®^Systems). The trough tank was partially covered to minimize visual disturbances of the fish, although dim light corresponding to the diel cycle remained present in the tank.

### Experimental procedures to measure specific dynamic action

Respirometry trials utilizing randomized paired control and omeprazole feedings were performed to determine acute effects of omeprazole on the metabolic rate of Nile tilapia. Eight Nile tilapia (19.5 ± 4.29 g) were used for the paired control and omeprazole feedings. The experiment started by weighing and placing the Nile tilapia into the individual respirometry chambers. The following 48 h were used for fasting and to collect ṀO_2_ data for estimates of SMR. The fish were then voluntarily fed meals of a commercial balanced diet (Bluewater Feed Company) equaling 2% BM via a feeding port. In a randomized fashion, half of the meals were dosed with omeprazole (TCI, Portland OR United States). Fish were fed voluntarily inside the respirometry chambers *via* a feeding port, eliminating the need for sham feedings for stress control (Chabot et al., 2016). Meals were consumed completely within 30 min. The metabolic rates of the fish were recorded for 96 h post-feeding, allowing the oxygen consumption rate to peak after feeding and then return to the SMR following the post-prandial response. Each fish was given both an omeprazole-dosed and a control sham treated meal over the course of the experiment to allow for pairwise comparison between the treatments in each individual fish.

SMR and SDA variables were calculated using the R package “FishMO2”, which uses non-parametric quantile regression to correct for circadian activity patterns when calculating SDA variables (Chabot et al., 2016). Tau (*t*) was set at 0.2 to calculate SMR and a lambda (λ) value of 24 h was used as the activity cycle variable as the standard protocol suggests. The R package was used to estimate SMR, peak time post feeding, postprandial peak, net postprandial peak, SDA duration and SDA magnitude of the individual fish. The variables were then compared between acute omeprazole and control feedings.

### Growth trial

To further understand the long-term effects on stomach acidification, growth rates of fish on control and omeprazole treatments were determined. To track individual growth rates all fish were anaesthetized with MS222 (1:10,000) and implanted with 8 mm PIT (passive integrated transponder) tags (Biomark, Boise, ID, United States) into the peritoneal cavity through an incision made with a #11 scalpel blade. Fish were allowed 1 week to recover. The growth trial was conducted using two groups of 23 Nile tilapia (n = 23) (33.0 ± 10.4 g) fed either a control feed, the commercial balanced diet (Bluewater Feed Company), or fed the control diet supplemented with omeprazole (TCI). All fish were kept at a constant water temperature of 26°C using a thermostat (InkBird Tech) and immersion heaters (300W Eheim Gmbh). The fish in both groups were fed manually at a rate of 2% BM per day separated over two meals. No differences were seen in feeding behaviors between treatments, and the entire ration was consumed at each feeding. The feeding trial was conducted for 8 weeks, with biometric parameters (standard length and mass) taken every 2 weeks, which allowed for adjustment of the ration size (2%) for the growing fish.

Over the 8 weeks, the BM data was compiled, and specific growth rate (SGR) was calculated as

$$SGR=\frac{\left(\ln M_{f}-\ln\;M_{i}\right)}{T}$$

where M_f_ is the final mass (g), M_i_ is the initial mass, and T is the time (days) between measurements. Fulton’s condition factor (K) (Fulton, 1904) was calculated as

$$K=\frac{{100,000}^{*}M}{L^{3}}$$

where M is the wet mass (g) and L is the corresponding standard length (mm) of the fish.

Growth rates and condition factor were calculated over the entire eight-week period to determine if growth rates were consistent over the feeding study. These measures were used to compare the increments of mass gain between different treatment groups based on growth per day (SGR). At the end of the trial, the tilapia were euthanized with an overdose of buffered MS222 (1:5000 w:v) followed by spinal transection, and fish mass, and standard length was recorded.

### Experimental procedures to measure gastric evacuation

A gastric evacuation trial was conducted using two groups of 60 Nile tilapia (46.2 ± 13.7 g) randomly assigned to one of the treatment groups (control or omeprazole). The daily feeding rate throughout the trial was 1% BM day^−1^. This meal size was the equivalent to the meal size given in the growth trial that was provided twice daily (2% × 1% BM). Serial dissection at five selected timepoints 1, 3-, 6-, 12-, and 24-h post-feeding (n = 10) was used to assess gastric contents gravimetrically. At each time point, tilapia from each group were euthanized with an overdose of buffered MS222 (1:5000 w:v) followed by spinal transection, and fish mass and standard length were recorded. The stomach of the fish was then excised, and all stomach contents were collected into pre-weighed tubes for mass determination. Stomach contents were then weighed, and frozen on dry ice for later dry matter analysis. Dry matter of the stomach contents was determined by drying the sample at 105°C until constant mass was reached (AOAC, 2000).

### Statistical analysis

Data is presented as means ± standard deviation (SD). Respirometry data (SMR, peak time post feeding, peak value, net peak, SDA duration and SDA magnitude) were analyzed using paired Student’s t-tests, while growth (SGR), chyme pH, and biometric (K) data were analyzed using a two-way analysis of variance (ANOVA) followed by a Student-Newman-Keuls (SNK) post hoc test. SGR and chyme pH data were transformed to satisfy conditions for a parametric test. Gastric evacuation and stomach (full, empty) and intestine pH data were either not normally distributed or lacked homogeneity of variance even after transformation and were therefore analyzed using two separate Kruskal Wallis one-way ANOVAs on ranks for control and omeprazole groups over time. When appropriate, either a Student’s t-test or Wilcoxon rank sum exact test was used to compare treatment differences at each time point. Results were considered significant at *p* ≤ 0.05. Sigmaplot 11.0 software was used for statistical analyses (Systat Software Inc., Palo Alto, CA).

## Results

**FIGURE 1:**
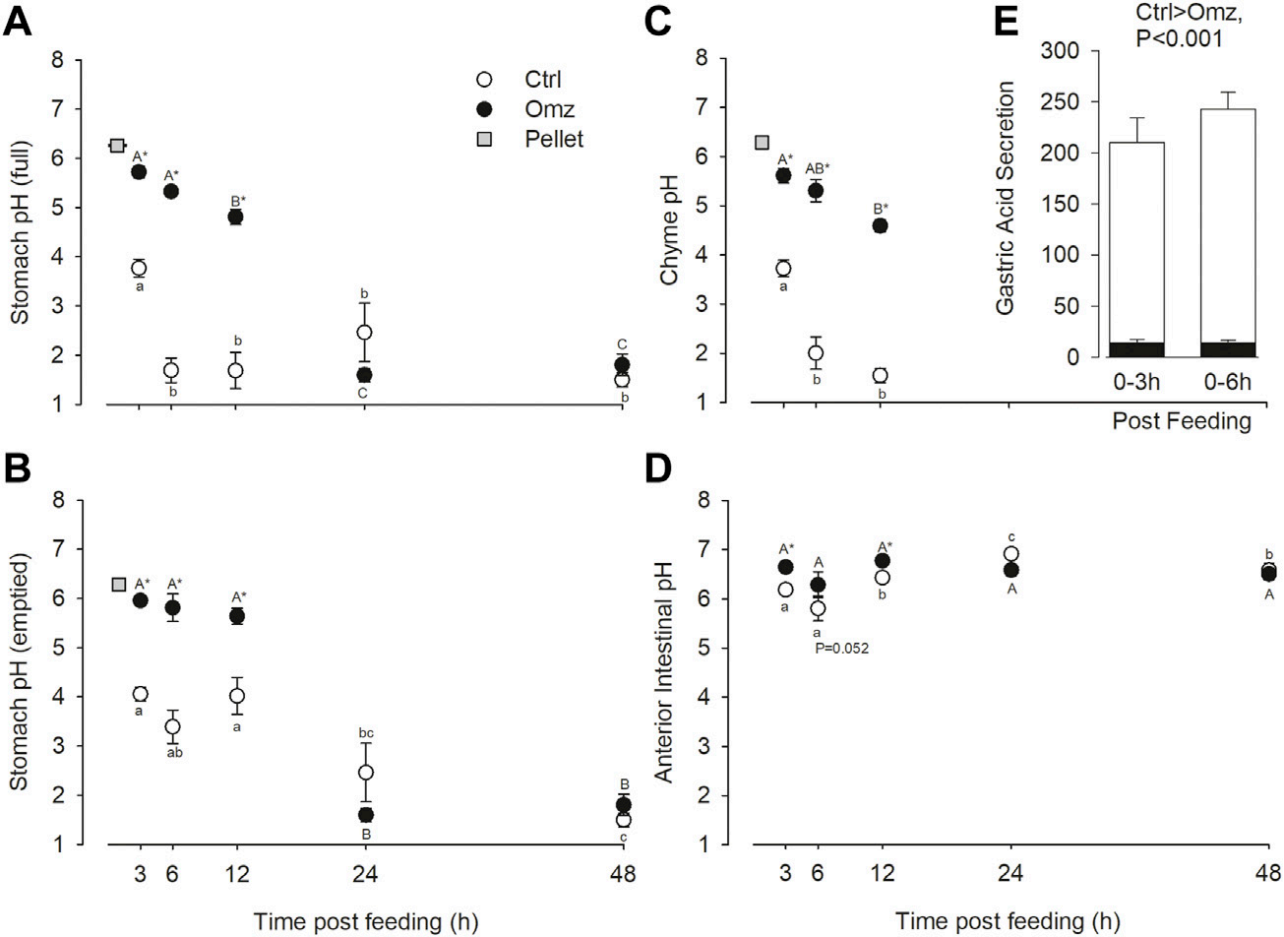
Nile tilapia (*Oreochromis niloticus*) gut pH measurements and gastric acid secretion rates. PIT tagged Nile tilapia were fed a 1% BM ration of pellets that were either sham treated (Control; white fill) or dosed with omeprazole at 25 mg kg^−1^ BM (Omeprazole; black fill) for 30 days. Tilapia were sampled at 3-h, 6-h, 12-h, 24-h and 48-h post-feed. The pH of the full stomach **(A)**, the emptied stomach (mucosa only) **(B)**, the isolated stomach chyme **(C)**, and the anterior intestine with contents **(D)** were measured. Note, stomach chyme was not present past 12 h post-feed, so no data is present at 24 and 48 h **(E)** Gastric acid secretion rates (µEq H^+^ g^−1^ feed h^−1^) were estimated over the 0–3 h and 0–6 h post prandial periods from pH change in **(A)** and the pellet titration curve (Supplementary Figure S1). Data were analyzed by two-way ANOVA with SNK post hoc test; n = 6. Time points with unshared letters are significantly different from each other. Asterisks signify significant differences between the two treatment groups.

The pellets fed to the fish had a starting pH of 6.261 ± 0.016 (n = 3). In control fish, at the first sampling point 3 h post feeding a 1% BM meal, the pH of the stomach was around pH 4 as measured in the stomach full or emptied, and the separated chyme (Figures 1A–C, respectively). From 6 h onward, the full stomach pH significantly declined further to less than pH 2. This was reflected in the stomach chyme readings but not the emptied stomach pH. In the omeprazole treatment group, stomach pH was significantly higher than the control fish from 3 to 12 h post feeding in the pH five to six range. By 24 h stomach pH was significantly lower and not different from the control fish. The patterns of full stomach pH changes in control and omeprazole groups reflected patterns seen in chyme pH rather than the emptied stomach. The calculated gastric acid secretion rates over the 0–3 h and 0–6 h post prandial periods were depressed by over 90% in the omeprazole treated fish compared to the sham controls (Figure 1E).

The pH in the anterior intestinal was lower 3–6 h post feeding compared to later time points in control fish, which reflects the input of the acidic chyme from the stomach and peaked at a higher pH of 7.073 at 24 h (Figure 1D). Only during the first 12 h post feeding was the intestinal pH in omeprazole treated fish higher than in controls. Note that the 6 h time point was marginally non-significant (*p* = 0.052). There were also no significant changes in intestinal pH in omeprazole treated fish over time.

**FIGURE 2:**
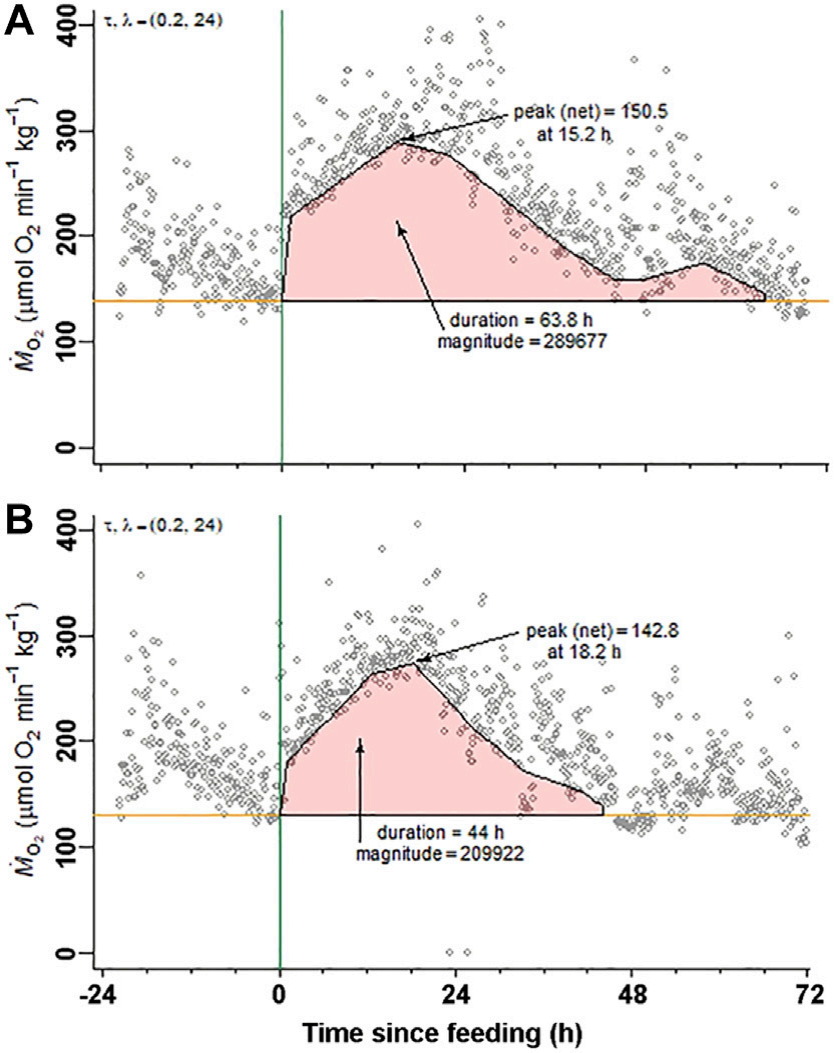
Nile tilapia (*Oreochromis niloticus*) Specific Dynamic Action measurements. A representative set of metabolic rate measurements (Ṁ_O2_; µmol O_2_ min^−1^ kg^−1^) over time from a Nile tilapia (19.5 g) following a 2% body mass (BM) voluntarily fed meal of **(A)** control pellets and **(B)** 25 mg kg^−1^ BM omeprazole dosed pellets. Standard metabolic rate (SMR) and specific dynamic action (SDA) parameters (time to peak, peak, net peak, duration, and magnitude) were calculated according to Chabot et al. (2016) using the R package “FishMO2”.

During the respirometry trial, tilapia voluntarily consumed the full meal provided in the respirometry chambers within 30 min. The SMR values measured in the 48 h period prior to feeding were not significantly different between the two treatment groups establishing a comparable baseline (Figure 2; Table 2). The SDA peak or net peak and time to peak values were not significantly different between the two treatment groups. Net peak data is not shown. However, the SDA duration in tilapia fed the omeprazole dosed meals was 34.4% shorter and the overall SDA magnitude or scope was 34.5% lower compared to the paired control meal.

**TABLE 2:** Standard metabolic rate (SMR) and Specific Dynamic Action (SDA) parameters measured in Nile tilapia (*Oreochromis niloticus*) voluntarily fed a meal of 2% BM of either sham-treated pellets (Control) or pellets treated with omeprazole at a final dose of 25 mg kg^−1^ BM. The variables were compared using paired t-tests n = 8.

Specific dynamic action variables					
Treatment	SMR (ṀO_2_)	Peak time (h)	Peak (ṀO_2_)	Duration (h)	SDA (x10^3^) (mg O_2_ kg^−1^)
Control	129.2 ± 5*.*2	12.1 ± 2*.*1	248.2 ± 9*.*0	63.8 ± 3*.*8	246 ± 28
Omeprazole	135.9 ± 9*.*6	15.2 ± 1*.*3	248.3 ± 11*.*4	41.8 ± 2*.*6	161 ± 25
p	0.556	0.192	0.992	0.002	0.015

**FIGURE 3:**
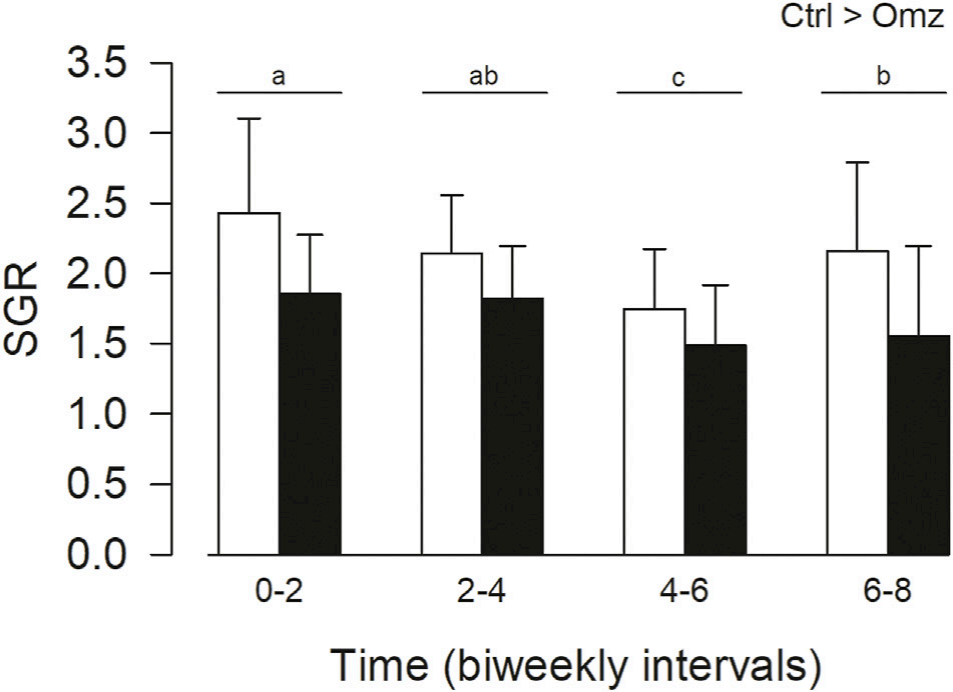
Nile tilapia (*Oreochromis niloticus*) growth trial. PIT tagged Nile tilapia were fed daily a 2% BM ration of pellets that were either sham treated (Control; Ctrl, white bars) or dosed with omeprazole at 25 mg kg^−1^ BM (Omeprazole; solid bars) over an eight-week period and weighed every 2 weeks. Specific growth rates (SGR) were calculated over bi-weekly intervals. Data was analyzed by two-way ANOVA with SNK post hoc test; n = 23. There were significant treatment (Ctrl>Omz) and time effects but without interactions. The time intervals with unshared letters are significantly different from each other.

The specific growth rates were calculated from the eight-week growth trial (Figure 3). Fish fed equally well on both diets, and rations were completely consumed during each feeding. Over the eight-week period the omeprazole group exhibited a significant reduction in SGR by 21.3% (Ctrl 2.12 ± 0.45; Omeprazole 1.67 ± 0.32 *p* < 0.001). There were also some time dependent changes in SGR over the eight-week period with a significant decrease in SGR over time with no interaction of time and treatment. At the end of the trial, the omeprazole associated decrease in growth rates resulted in a lower total biomass gain of 502.65 g (n = 23) or an average of 20 g less mass added per individual, in comparison to the control group. There was no significant difference in condition factor during the feeding trial (Ctrl 2.39 ± 0.53; Omeprazole 2.35 ± 0.52, *p* = 0.819).

**FIGURE 4:**
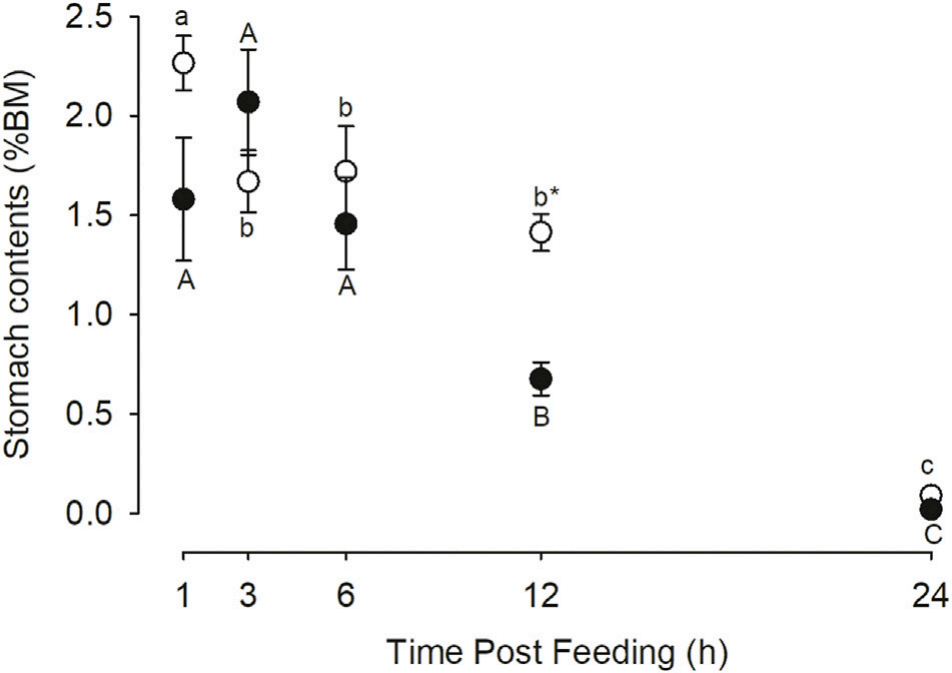
Nile tilapia (*Oreochromis niloticus*) stomach evacuation. PIT tagged Nile tilapia were fed a 1% BM ration of pellets that were either sham treated (Control; white circles) or dosed with omeprazole at 25 mg kg^−1^ BM (Omeprazole; black circles) for 30 days. Serial dissections at five selected timepoints 1, 3-, 6-, 12-, and 24-h post-feeding (n = 10) was used to assess gastric contents gravimetrically. These values represent the wet mass of stomach contents as a percentage of animal body mass. Significant differences between treatments were only found at the 12-h post-feeding timepoint (*p* < 0.05). Time points with unshared letters are significantly different from each other.

The gastric evacuation data is presented in Figure 4. In the control fish there was a decrease in stomach content wet mass after 1 h post feeding which was relatively stable until 12 h but by 24 h the stomach was essentially empty. In the case of the omeprazole treated fish, the amount of stomach contents did not change over the first 6 h post feeding but had decreased significantly by 12 h and was essentially absent by 24 h. At the 12 h time point there was significantly less chyme in the stomach of the omeprazole treated fish compared to the control (*p* = 0.013), indicating a more rapid gastric evacuation.

## Discussion

The gastric proton pump inhibitor omeprazole predictably inhibited post-prandial gastric acidification in juvenile Nile tilapia. This greater than 90% inhibition of acidification corresponded with a decrease in both SDA duration and magnitude. It followed that there was a more rapid gastric evacuation or emptying following a meal. Although there was a decrease in the magnitude of the SDA, this was not reflected in a digestive energy savings that could then be allocated to increased growth but rather a reduction in the SGR by more than 20% was observed in the omeprazole treated fish. Collectively, these data suggest that simulation of an agastric phenotype in fish by pharmacological “knock down” of the gastric proton pump with omeprazole may be constrained by poor digestion, which highlights the high degree of conservation of the gastric phenotype in vertebrates. This could imply that the emergence of the agastric phenotype in fishes is correlated with the availability of easily digestible diets, but this hypothesis requires future testing.

The inhibition of gastric acidification with omeprazole was consistent with earlier studies (*Squalus acanthias* Wood et al., 2009; *Boa constrictor*, Andrade et al., 2004). The patterns of pH changes indicated that the pH of the full stomach was largely reflective of the chyme pH rather than the gastric mucosa (emptied stomach). We also estimated gastric acid secretion from the pH changes of the stomach contents and estimated buffer capacity of 1 g of pellets which we predicted would be relatively stable over the 6 h post prandial period based on our gastric evacuation data set. In control fish, 210–240 µEq H^+^ were secreted to acidify 1 g of pellets. In contrast omeprazole treated fish only secreted 14 µEq H^+^, representing a >90% decrease in acid secretion. The degree of inhibition is in keeping with human studies, where omeprazole doses of 20–40 mg resulted in 80%–100% reductions in stimulated acid secretion (Olbe et al., 1989). It followed that the chyme in the anterior intestine was significantly less acidic in omeprazole treated fish. This reduction in the acidity of the intestinal chyme might be an explanation for the increased gastric evacuation rate observed. It is known that gastric evacuation or emptying is under neuro-endocrine control, and that enteroendocrine cells in the anterior intestine can delay gastric emptying by secreting “brake” hormones such as cholecystokinin (CCK) in response to acidic chyme entering the intestine (Olsson et al., 1999; Goyal et al., 2019). However, future work should confirm if CCK release by enteroendocrine cells is decreased in omeprazole treated fish to validate this hypothesis.

The SDA represents the total energy expended on the ingestion, digestion, absorption, and assimilation of the meal (reviewed by McCue 2006; Secor 2009; Wang and Rindom 2021). The present study revealed a decrease in both SDA duration and magnitude with omeprazole treatment, suggesting that the meals were digested and assimilated in a shorter period and were evacuated from the alimentary canal more rapidly. This observed decrease in total duration was unexpected, as we predicted that inhibition of gastric acidification would retard digestion and thus prolong the duration of digestion and assimilation. This contrasts with mammalian studies, in which omeprazole did delay gastric emptying following a solid meal (Benini et al., 1996; Sanaka et al., 2010). However, this clearly did not happen in tilapia and was confirmed by the shorter stomach residency time in the omeprazole-treated fish. The amount of chyme in the stomach was not different between treatments at the earlier time points (1–6-h) but by 12 h post-feeding the omeprazole treated group only had half as much chyme left compared to the control. No detectable difference was seen at 24-h post-feeding as the stomach in both treatment groups was almost completely emptied. In fish exposed to low temperature, hypoxia or hypercapnia, an increase in SDA duration has been observed (Jordan and Steffensen 2007; Tirsgaard et al., 2015a, b). However, these variables were controlled in the present study and cannot account for the observed changes.

There was no significant difference in the time to peak or peak of the SDA suggesting there was no detectable direct impact of H^+^/K^+^-ATPase inhibition directly on ṀO_2_. However, it cannot be rule out that there could be other oxygen consuming processes masking these changes such as intestinal processes acting sooner because of the earlier gastric evacuation. The significant decrease in SDA magnitude indicates that there should be either additional energy available for growth since it is not being partitioned toward digestion, and/or there is simply less digestion occurring overall. Significantly the growth trial data revealed a decrease in the SGR of omeprazole-treated fish. The reduced SDA duration and magnitude can be explained by a decrease in nutrient absorption and/or assimilation resulting in reduced growth when compared to the control group. Previous work in channel catfish (Brown and Cameron 1991a, b) and Burmese pythons (McCue et al., 2005) have shown that the SDA can be mimicked by an infusion of amino acids and that this increase can be significantly blocked by the protein synthesis inhibitor cycloheximide. These results indicate the major contribution of protein assimilation to SDA. Future digestibility trials will address whether protein assimilation has indeed been adversely impacted by omeprazole. The use of cycloheximide in Nile tilapia can also be used to confirm the SDA response.

Dietary acidification is an approach that has been used to help understand the cost of gastric acid secretion in barramundi *Lates calcarifer* (Goodrich et al., 2022). The SDA response of the animals after being fed an acidified diet (from pH 5.82 to 4.09) showed a significant reduction in the overall magnitude of the SDA response by ∼45% in comparison to the control group fed a 3.5% ration (Goodrich et al., 2022), which is similar to the observed decreases in the current study (34.5%). Along with the alterations to SDA response, a significant reduction in the alkaline tide response was observed, with blood pH and bicarbonate not showing significant increases post feeding seen in control fish. In dogfish fed a 2% ration, Wood et al. (2009) found a reduction in the alkaline tide using omeprazole. Citing a previous study in rainbow trout where dietary acidification resulted in decreased mRNA expression of *atp4a* (Sugiura 2006), Goodrich et al. (2022) suggested that the reduction in energy expenditure may be in part due to a similar phenomenon occurring in the fish during their trial. Future studies on SDA response with omeprazole treatment in teleost fishes should be done observing the alkaline tide for more clear comparisons of the results with Goodrich et al. (2022).

In the study of Andrade et al. (2004) in the snake *B. constrictor*, although omeprazole inhibited the alkaline tide caused by gastric HCO_3_^−^ secretion into the blood, it had no significant effect on the SDA unlike in the present study. In addition to taxonomic (fish versus reptile) and trophic (frequent small meals versus infrequent large meals) differences, the discrepancy with the present study can be at least partially explain by differences in experimental design. In the present study on tilapia, a repeated measures design allowed for individuals to serve as their own controls and thus provided greater statistical power to detect differences against a background of inter-individual variation. Goodrich et al. (2022) took the same repeated measures approach in their barramundi SDA study with similar successful results in detecting treatment differences. Notably, we were unable to detect differences if we used an unpaired *t*-test (unpublished observations). Also, the *B. constrictor* study made use of two groups of animals, that unfortunately had differing SMRs that could potentially complicate the interpretation of results as well (Andrade et al., 2004). However, more recent data in *B. constrictor* indicates that the contribution of the gastric proton pump to SDA is negligible since buffering the meal with either carbonate or bone meal, which increase acid secretion, did not alter SDA (Henriksen et al., 2015; Nørgaard et al., 2016) and that pyloric ligation abolished SDA (Enok et al., 2013). The latter study on pyloric ligation indicates that post-gastric process are the main contributors to SDA consistent with the findings with cycloheximide discussed earlier (Brown and Cameron 1991a, b; McCue et al., 2005).

In the present study, we found the voluntary feeding within the respirometry chamber was important to the success of the SDA measurements. Repeated force feeding by intubation was problematic resulting in regurgitation, and markedly elevated ṀO_2_ (MR and JMW, unpublished observations; Chabot et al., 2016). In addition, the required dilution of the ground moist pellets for liquid delivery *via* the feeding cannula necessitated delivery of a smaller meal size (<2% BM) since tilapia have a small stomach (Morrison and Wright 1999). We were also able to feed repeated meals allowing for repeated measures statistical analysis.

The growth rates we report are within the range of values reported in other studies with *O. niloticus* (e.g., Makori et al., 2017). The reduced growth in the omeprazole-treated fish could be explained by decreased protein digestion and absorption from the feed, as acid denaturation is the first step in protein digestion in gastric fish species (Bakke et al., 2010). While gastric pH plays a small role in denaturing structural bonds in proteins, the importance lies in the activation of pepsin for protein digestion (Sanny et al., 1975; Kageyama 2002). Gastric acid digestion increases the amount of soluble polypeptide that get broken down into di- and oligo-peptides, increasing the speed of intestinal absorption of dietary amino acids (Grabner and Hofer 1989; Hamdan et al., 2009). Pepsin cleaves off di- and oligo-peptides at preferential bonds between hydrophobic and aromatic amino acids including phenylalanine, tyrosine, and tryptophan (Kageyama 2002). As pepsin is one of the three main proteases in the digestive tract, along with intestinal trypsin and chymotrypsin, this decrease in digestive function of the omeprazole treated fish may prevent proper breakdown of protein, leading to reduced absorption (Kageyama 2002; Yúfera and Darías, 2007). With the omeprazole treatment and resultant suppression of stomach acidification, protein digestion occurring in the tilapia gut may have been reduced as less pepsinogen was activated, which may have resulted in reduced time in the intestine for absorption and decreased overall digestive transit times. However, it has yet to be determined if the latter has been affected by omeprazole treatment and future work is required. Omeprazole treatment did not appear to influence feeding behavior or appetite, as Nile tilapia are voracious eaters and readily took up the pellets during feeding. This behavior did not change during the course of the trial with complete meal consumption observed.

A key requirement for growth in fish species is adequate digestible phosphorus from the diet. The reduction in growth observed in the present study could in part be caused by a decrease in phosphorus uptake. Phosphorus is a very important nutrient for fish, as it is directly involved in all energy related processes involving ATP and is an essential component of cell membranes and nucleic acids in addition to its obvious role in bone growth and remodeling (National Research Council, 2011; Hossain and Yoshimatsu, 2014). As phosphorus is scarce in fresh water, the diet is the most important source, and digestible forms are essential to maintain health of the fish. The main form of dietary phosphorus in fish feeds is hydroxyapatite or bone phosphate in fish meal, which requires strong acidity in order to be adequately solubilized for intestinal absorption (Cho and Bureau, 2001; Sugiura, 2006; Hua and Bureau, 2010). This observation coupled with the inhibition of acid production in the omeprazole treated fish in the current study emphasizes the importance of determining the digestibility of this key nutrient moving forward.

In humans, omeprazole is widely used in the chronic treatment of gastric ulcers and gastroesophageal reflux disease (GERD) (Schubert 2017). Although clinical studies have shown that omeprazole can delay overall protein assimilation and potentially cause protein malabsorption (Evenepoel et al., 1998), which would be consistent with the present study in tilapia, there are generally negligible impacts from a nutritional perspective (Evenepoel et al., 1998; Gibbons and Gold 2003). This discrepancy can be explained by the use of restricted ration (2% BM day^−1^) in the present study, which is generally not a factor in the western diet. Thus, in humans, malabsorption would be masked by a dietary excess in nutrients.

## Conclusions and future perspectives

The use of omeprazole was effective in altering digestion in the treated group in both acute feeding post-prandial metabolic rate (i.e., SDA variables) and over the course of the long-term growth and gastric evacuation trials. The results indicate that omeprazole impaired the production of gastric acid (achlorhydria) and potentially the activation of pepsin in the stomach of these fish, significantly compromising gastric function to the point where they could be considered “functionally agastric”. The omeprazole treatment offers a novel way to explore the metabolic expense of the acidic environment of the stomach. This study paves the way for future studies to address the nature of the effects of gastric acid inhibition on growth, specifically examining protein digestibility and assimilation and the microbiome. Since the stomach also acts as a barrier for pathogen entry into the intestine, future work can also address this role in enteric disease susceptibility in fishes.

## Data Availability

The raw data supporting the conclusions of this article will be made available by the authors, without undue reservation.

## References

[B1] Andrade D. V. De Toledo L. P. Abe A. S. Wang T. (2004). Ventilatory compensation of the alkaline tide during digestion in the snake *Boa constrictor* . J. Exp. Biol. 207, 1379–1385. 10.1242/jeb.00896 15010489

[B2] AOAC (Association of Analytical Chemists) (2000). Official methods of analysis of the association of official analytical chemists. 14th ed. Rockville, MD, USA: Association of Analytical Chemists.

[B3] Bakke A. M. Glover C. Krogdahl Å. (2010). “Feeding, digestion, and absorption of nutrients,” in Fish physiology: The multifunctional gut of fish. Editors Grosell M. Farrell A. Brauner C. (Academic Press), 57–110.

[B4] Baktoft H. Jacobsen L. Skov C. Koed A. Jepsen N. Berg S. (2016). Phenotypic variation in metabolism and morphology correlating with animal swimming activity in the wild: Relevance for the OCLTT (oxygen- and capacity-limitation of thermal tolerance), allocation and performance models. Conserv. Physiol. 4, cov055–14. 10.1093/conphys/cov055 27382465 PMC4922247

[B5] Barnawi E. A. Doherty J. E. Ferreira P. G. Wilson J. M. (2020). Extra-gastric expression of the proton pump H^+^/K^+^-ATPase in the gills and kidney of the teleost *Oreochromis niloticus* . J. Exp. Biol. 223, jeb214890. 10.1242/jeb.214890 32611790

[B6] Barrington E. J. W. (1942). Gastric digestion in the lower vertebrates. Biol. Rev. 17, 1–27. 10.1111/j.1469-185x.1942.tb00429.x

[B7] Benini L. Castellani G. Bardelli E. Sembenini C. Brentegani M. T. Caliari S. (1996). Omeprazole causes delay in gastric emptying of digestible meals. Dig. Dis. Sci. 41, 469–474. 10.1007/BF02282320 8617117

[B8] Brown C. R. Cameron J. N. (1991a). The induction of specific dynamic action in channel catfish by infusion of essential amino acids. Physiol. Zool. 64, 276–297. 10.1086/physzool.64.1.30158524

[B9] Brown C. R. Cameron J. N. (1991b). The relationship between specific dynamic action (SDA) and protein synthesis rates in the channel catfish. Physiol. Zool. 64, 298–309. 10.1086/physzool.64.1.30158525

[B10] Castro L. F. C. Goncalves O. Mazan S. Tay B.-H. Venkatesh B. Wilson J. M. (2014). Recurrent gene loss correlates with the evolution of stomach phenotypes in gnathostome history. Roy. Soc. B. 281, 20132669–9. 10.1098/rspb.2013.2669 PMC3866411 24307675

[B11] Castro L. F. C. Lopes-Marques M. Gonçalves O. Wilson J. M. (2012). The evolution of pepsinogen C genes in vertebrates: Duplication, loss and functional diversification. PloS one 7, e32852. 10.1371/journal.pone.0032852 22427897 PMC3298455

[B12] Chabot D. Koenker R. Farrell A. P. (2016). The measurement of specific dynamic action in fishes. J. Fish. Biol. 88, 152–172. 10.1111/jfb.12836 26768974

[B13] Chakraborty S. C. Ross L. G. Ross B. (1995). Energy budget and metabolism in common carp, *Cyprinus carpio* L., fed on different dietary protein levels and at different ration levels. Aquac. Nutr. 1, 179–187. 10.1111/j.1365-2095.1995.tb00042.x

[B14] Cho C. Y. Bureau D. P. (2001). A review of diet formulation strategies and feeding systems to reduce excretory and feed wastes in aquaculture. Aquac. Res. 32, 349–360. 10.1046/j.1355-557x.2001.00027.x

[B15] Cho C. Y. (1992). Feeding systems for rainbow trout and other salmonids with reference to current estimates of energy and protein requirements. Aquaculture 100, 107–123. 10.1016/0044-8486(92)90353-m

[B16] Enok S. Simonsen L. S. Wang T. (2013). The contribution of gastric digestion and ingestion of amino acids on the postprandial rise in oxygen consumption, heart rate and growth of visceral organs in pythons. Comp. Biochem. Physiol. A Mol. Integr. Physiol. 165A, 46–53. 10.1016/j.cbpa.2013.01.022 23384684

[B17] Evenepoel P. Claus D. Geypens B. Maes B. Hiele M. Rutgeerts P. (1998). Evidence for impaired assimilation and increased colonic fermentation of protein, related to gastric acid suppression therapy. Aliment. Pharmacol. Ther. 12, 1011–1019. 10.1046/j.1365-2036.1998.00377.x 9798807

[B18] Fulton T. W. (1904). The rate of growth of fishes. 22^nd^ Annu. Rep. Fish. Board Scotl., 141–241.

[B19] Gibbons T. E. Gold B. D. (2003). The use of proton pump inhibitors in children: A comprehensive review. Paediatr. Drugs 5, 25–40. 10.2165/00128072-200305010-00003 12513104

[B20] Goodrich H. R. Wilson R. W. Smullen R. Barnes A. C. Franklin C. E. (2022). Acidified fish feeds reduce the energetic and physiological costs of digestion in juvenile barramundi (*Lates calcarifer*). Aquaculture 546, 737400–737414. 10.1016/j.aquaculture.2021.737400

[B21] Goyal R. K. Guo Y. Mashimo H. (2019). Advances in the physiology of gastric emptying. Neurogastroenterol. Motil. 31, e13546. 10.1111/nmo.13546 30740834 PMC6850045

[B22] Grabner M. Hofer R. (1989). Stomach digestion and its effect upon protein hydrolysis in the intestine of rainbow trout (*Salmo gairdneri Richardson*). Comp. Biochem. Physiol. Part A Physiol. 92, 81–83. 10.1016/0300-9629(89)90745-7

[B23] Hamdan M. Moyano F. J. Schuhardt D. (2009). Optimization of a gastrointestinal model applicable to the evaluation of bioaccessibility in fish feeds. J. Sci. Food Agric. 89, 1195–1201. 10.1002/jsfa.3574

[B24] Heidelbaugh J. J. (2013). Proton pump inhibitors and risk of vitamin and mineral deficiency: Evidence and clinical implications. Ther. Adv. Drug Saf. 4, 125–133. 10.1177/2042098613482484 25083257 PMC4110863

[B25] Henriksen P. S. Enok S. Overgaard J. Wang T. (2015). Food composition influences metabolism, heart rate and organ growth during digestion in Python regius. Comp. Biochem. Physiol. A Mol. Integr. Physiol. 183, 36–44. 10.1016/j.cbpa.2014.12.031 25553896

[B26] Hopkins K. D. (1992). Reporting fish growth: A review of the basics. J. World Aquac. Soc. 23, 173–179. 10.1111/j.1749-7345.1992.tb00766.x

[B27] Hossain M. A. Yoshimatsu T. (2014). Dietary calcium requirement in fishes. Aquac. Nutr. 20 (1), 1–11. 10.1111/anu.12135

[B28] Hua K. Bureau D. P. (2010). Quantification of differences in digestibility of phosphorus among cyprinids, cichlids, and salmonids through a mathematical modelling approach. Aquaculture 308 (3–4), 152–158. 10.1016/j.aquaculture.2010.07.040

[B29] Jobling M. (1981). The influences of feeding on the metabolic rate of fishes: A short review. J. Fish. Biol. 18, 385–400. 10.1111/j.1095-8649.1981.tb03780.x

[B30] Jordan A. D. Steffensen J. F. (2007). Effects of ration size and hypoxia on specific dynamic action in the cod. Physiol. Biochem. Zool. 80, 178–185. 10.1086/510565 17252514

[B31] Kageyama T. (2002). Pepsinogens, progastricsins, and prochymosins: Structure, function, evolution, and development. Cell. Mol. Life Sci. 59, 288–306. 10.1007/s00018-002-8423-9 11915945 PMC11146132

[B32] Koelz H. R. (1992). Gastric acid in vertebrates. Scand. J. Gastroenterol. Suppl. 27, 2–6. 10.3109/00365529209095998 1290053

[B33] Lindberg P. Brändström A. Wallmark B. (1987). Structure-activity relationships of omeprazole analogues and their mechanism of action. Trends Pharmacol. Sci. 8, 399–402. 10.1016/0165-6147(87)90107-6

[B34] Lindberg P. Nordberg P. Alminger T. Brandstrom A. Wallmark B. (1986). The mechanism of action of the gastric acid secretion inhibitor omeprazole. J. Med. Chem. 29, 1327–1329. 10.1021/jm00158a001 3016260

[B35] Lobel P. S. (1981). Trophic biology of herbivorous reef fishes: Alimentary pH and digestive capabilities. J. Fish. Biol. 19, 365–397. 10.1111/j.1095-8649.1981.tb05842.x

[B36] Makori A. J. Abuom P. O. Kapiyo R. Anyona D. N. Dida G. O. (2017). Effects of water physico-chemical parameters on tilapia (*Oreochromis niloticus*) growth in earthen ponds in Teso North Sub-County, Busia County. Fish. Aquat. Sci. 20, 30. 10.1186/s41240-017-0075-7

[B37] McCue M. D. Bennett A. F. Hicks J. W. (2005). The effect of meal composition on specific dynamic action in Burmese pythons (*Python molurus*). Physiol. Biochem. Zool. 78, 182–192. 10.1086/427049 15778938

[B38] McCue M. D. (2006). Specific dynamic action: A century of investigation. Comp. Biochem. Physiol. A Mol. Integr. Physiol. 144, 381–394. 10.1016/j.cbpa.2006.03.011 16716621

[B39] McTavish D. Buckley M. M.-T. Heel R. C. (1991). Omeprazole. An updated review of its pharmacology and therapeutic use in acid-related disorders. Omeprazole. Drugs 42, 138–170. 10.2165/00003495-199142010-00008 1718683

[B40] Morii M. Takata H. Takeguchi N. (1989). Acid activation of omeprazole in isolated gastric vesicles, oxyntic cells, and gastric glands. Gastroenterology 96, 1453–1461. 10.1016/0016-5085(89)90512-x 2541041

[B41] Morrison C. M. Wright J. R. Jr (1999). A study of the histology of the digestive tract of the Nile tilapia. J. Fish. Biol. 54, 597–606. 10.1111/j.1095-8649.1999.tb00638.x

[B42] National Research Council (2011). Nutrient requirements of fish and shrimp. Washington, DC: National Academies Press. 10.17226/13039

[B43] Nelson J. A. (2016). Oxygen consumption rate v. rate of energy utilization of fishes: A comparison and brief history of the two measurements. J. Fish. Biol. 88, 10–25. 10.1111/jfb.12824 26768970

[B44] Nørgaard S. Andreassen K. Malte C. L. Enok S. Wang T. (2016). Low cost of gastric acid secretion during digestion in ball pythons. Comp. Biochem. Physiol. A Mol. Integr. Physiol. 194, 62–66. 10.1016/j.cbpa.2016.01.003 26802791

[B45] Olbe L. Cederberg C. Lind T. Olausson M. (1989). Effect of omeprazole on gastric acid secretion and plasma gastrin in man. Scand. J. Gastroenterol. Suppl. 24, 27–32. 10.3109/00365528909091240 2690329

[B46] Olsson C. Aldman G. Larsson A. Holmgren S. (1999). Cholecystokinin affects gastric emptying and stomach motility in the rainbow trout *Oncorhynchus mykiss* . J. Exp. Biol. 202, 161–170. 10.1242/jeb.202.2.161 9851905

[B47] Priede I. G. (1985). “Metabolic scope in fishes,” in Fish energetics: New perspectives. Editors Tytler P. Calow P. (London: Croom Helm), 33–64.

[B48] Rosewarne P. J. Wilson J. M. Svendsen J. C. (2016). Measuring maximum and standard metabolic rates using intermittent flow respirometry: A student laboratory investigation of aerobic metabolic scope and environmental hypoxia in aquatic breathers. J. Fish. Biol. 88, 265–283. 10.1111/jfb.12795 26768978

[B49] Sanaka M. Yamamoto T. Kuyama Y. (2010). Effects of proton pump inhibitors on gastric emptying: A systematic review. Dig. Dis. Sci. 55, 2431–2440. 10.1007/s10620-009-1076-x 20012198

[B50] Sanny C. G. Hartsuck J. A. Tang J. (1975). Conversion of pepsinogen to pepsin. Further evidence for intramolecular and pepsin-catalyzed activation. J. Biol. Chem. 250, 2635–2639. 10.1016/s0021-9258(19)41649-9 235522

[B51] Schubert M. L. (2017). Physiologic, pathophysiologic, and pharmacologic regulation of gastric acid secretion. Curr. Opin. Gastroenterol. 33, 430–438. 10.1097/MOG.0000000000000392 28787289

[B52] Schwartzbach A. Behrens J. Svendsen J. (2020). Atlantic cod *Gadus morhua* save energy on stone reefs: Implications for the attraction versus production debate in relation to reefs. Mar. Ecol. Prog. Ser. 635, 81–87. 10.3354/meps13192

[B53] Secor S. M. (2003). Gastric function and its contribution to the postprandial metabolic response of the Burmese python *Python molurus* . J. Exp. Biol. 206, 1621–1630. 10.1242/jeb.00300 12682094

[B54] Secor S. M. (2009). Specific dynamic action: A review of the postprandial metabolic response. J. Comp. Physiol. B 179, 1–56. 10.1007/s00360-008-0283-7 18597096

[B55] Smit H. (1968). Gastric secretion in the lower vertebrates and birds. In Handbook of Physiology section 6 alimentary canal vol. V bile, digestion, ruminal physiology (Code C. F., ed.), pp. 2791–2805.

[B56] Steffensen J. F. (1989). Some errors in respirometry of aquatic breathers: How to avoid and correct for them. Fish. Physiol. Biochem. 59, 49–59. 10.1007/BF02995809 24226899

[B57] Sugiura S. H. Roy P. K. Ferraris R. P. (2006). Dietary acidification enhances phosphorus digestibility but decreases H^+^, K^+^-ATPase expression in rainbow trout. J. Exp. Biol. 209, 3719–3728. 10.1242/jeb.02436 16985189

[B58] Svendsen J. C. Tirsgaard B. Cordero G. A. Steffensen J. F. (2015). Intraspecific variation in aerobic and anaerobic locomotion: Gilthead sea bream (*Sparus aurata*) and Trinidadian guppy (*Poecilia reticulata*) do not exhibit a trade-off between maximum sustained swimming speed and minimum cost of transport. Front. Physiol. 6, 43–12. 10.3389/fphys.2015.00043 25741285 PMC4330683

[B59] Taylor J. R. Grosell M. (2009). The intestinal response to feeding in seawater gulf toadfish, *Opsanus beta*, includes elevated base secretion and increased epithelial oxygen consumption. J. Exp. Biol. 212, 3873–3881. 10.1242/jeb.034579 19915130

[B60] Tirsgaard B. Moran D. Steffensen J. F. (2015). Prolonged SDA and reduced digestive efficiency under elevated CO_2_ may explain reduced growth in Atlantic cod (*Gadus morhua*). Aquat. Toxicol. 158, 171–180. 10.1016/j.aquatox.2014.11.009 25438123

[B61] Tirsgaard B. Svendsen J. C. Steffensen J. Fl. (2015). Effects of temperature on specific dynamic action in the Atlantic cod *Gadus morhua* . Fish. Physiol. Biochem. 41, 41–50. 10.1007/s10695-014-0004-y 25343877

[B62] Wang T. Rindom E. (2021). The physiological response to digestion in snakes: A feast for the integrative physiologist. Comp. Biochem. Physiol. A Mol. Integr. Physiol. 254, 110891. 10.1016/j.cbpa.2020.110891 33400953

[B63] Wilson J. M. Castro L. F. C. (2010). Morphological diversity of the gastrointestinal tract in fishes. Fish. Physiol. 30, 1–55.

[B64] Wood C. M. Schultz A. G. Munger R. S. Walsh P. J. (2009). Using omeprazole to link the components of the post-prandial alkaline tide in the spiny dogfish, *Squalus acanthias* . J. Exp. Biol. 212, 684–692. 10.1242/jeb.026450 19218520

[B65] Yúfera M. Darías M. J. (2007). Changes in the gastrointestinal pH from larvae to adult in Senegal sole (*Solea senegalensis*). Aquaculture 267, 94–99. 10.1016/j.aquaculture.2007.02.009

