# Supplementary material for: Inhibition of gastric acid secretion with omeprazole affects fish specific dynamic action and growth rate: Implications for the development of phenotypic stomach loss
Source: Front Physiol. 2022 Sep 27;13:966447. doi: 10.3389/fphys.2022.966447 (PMC9552000; doi:10.3389/fphys.2022.966447)
Supplement: Supplementary file 1 [file DataSheet1.PDF]

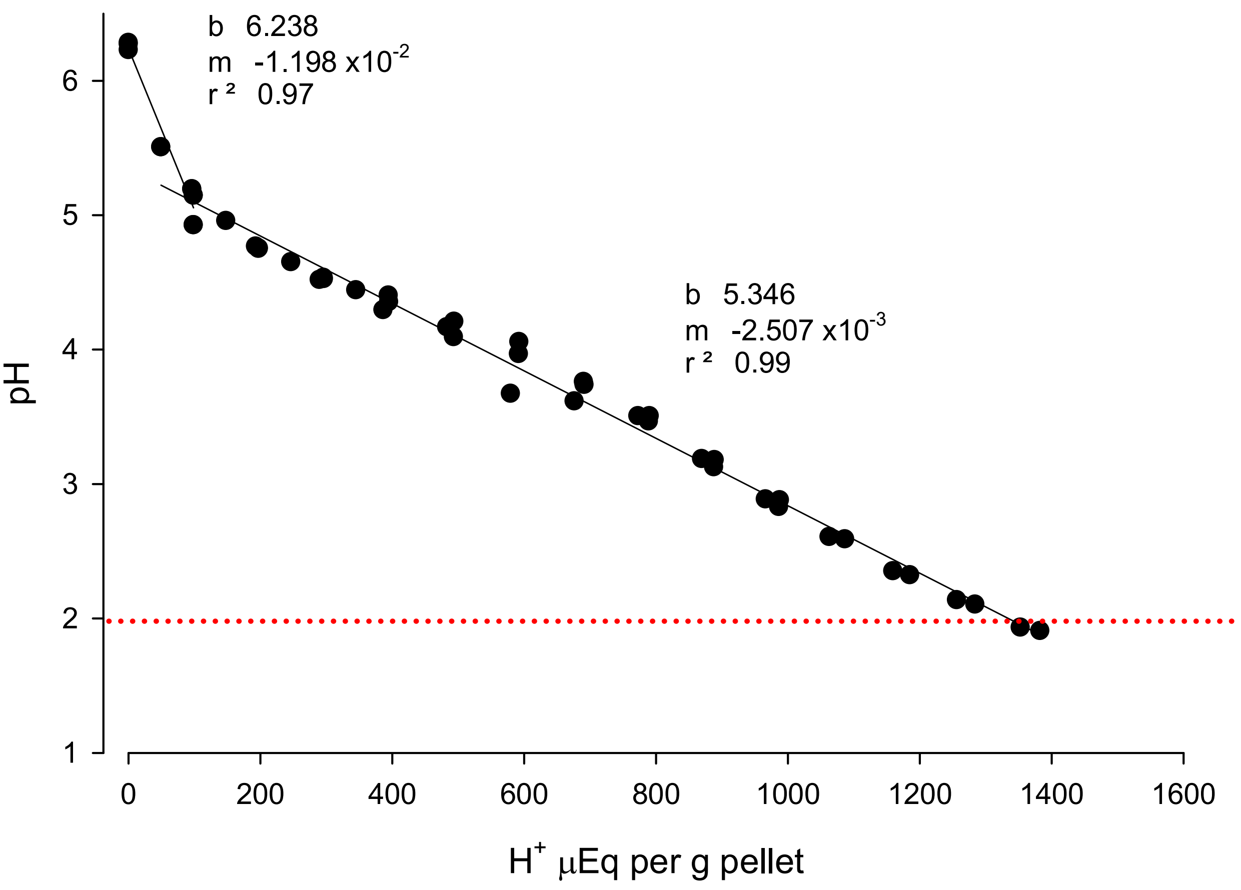

Figure S1. Titration of 1 g of pulverized fish feed pellets with 1 N HCl from an initial pH of 6.261 to below pH 2 (red dotted line). Linear regressions were fitted to the data over approximate pH ranges of 6.2-5.0 and 5.0-2.0. n=3

The following equations were used to estimate acid secretion (x) using changes in gastric pH measurements in Fig.1A from an initial pellet pH of 6.261 (pH_{pellet}-pH_{st}) and a 1% BM ration (1 g pellet per 100 g fish).

pH Range 6.2-5.0

Gastric acid secreted = [(pH_{pellet}-pH_{st}) - 6.238] / -0.01198

pH Range < 5.0

Gastric acid secreted = [(pH_{pellet}-pH_{st}) - 5.346] / -0.002507
